# Chronic Stress-Induced Elevation of Melanin-Concentrating Hormone in the Locus Coeruleus Inhibits Norepinephrine Production and Associated With Depression-Like Behaviors in Rats

**DOI:** 10.1093/ijnp/pyad069

**Published:** 2023-12-22

**Authors:** Nurhumar Kurban, Yu Qin, Hui-Ling Zhao, Xiao Hu, Xi Chen, Yi-Yi Zhao, Yu-Shuo Peng, Hong-Bo Wang, Su-Ying Cui, Yong-He Zhang

**Affiliations:** Department of Pharmacology, Peking University, School of Basic Medical Science, Beijing, China; Department of Pharmacology, Peking University, School of Basic Medical Science, Beijing, China; Department of Pharmacology, Peking University, School of Basic Medical Science, Beijing, China; Department of Pharmacology, Peking University, School of Basic Medical Science, Beijing, China; Department of Pharmacology, Peking University, School of Basic Medical Science, Beijing, China; Department of Pharmacology, Peking University, School of Basic Medical Science, Beijing, China; Department of Pharmacology, Peking University, School of Basic Medical Science, Beijing, China; Key Laboratory of Molecular Pharmacology and Drug Evaluation, Ministry of Education, Yantai University, Yantai, China; Department of Pharmacology, Peking University, School of Basic Medical Science, Beijing, China; Key Laboratory of Molecular Pharmacology and Drug Evaluation, Ministry of Education, Yantai University, Yantai, China; Department of Pharmacology, Peking University, School of Basic Medical Science, Beijing, China

**Keywords:** Melanin-concentrating hormone, MCHR1, depression-like behaviors, locus coeruleus, norepinephrine

## Abstract

**Background:**

Melanin-concentrating hormone (MCH) is a hypothalamic neuropeptide that projects throughout the central nervous system, including the noradrenergic locus coeruleus (LC). Our previous study suggested that MCH/MCH receptor 1 (MCHR1) in the LC may be involved in the regulation of depression. The present study investigated whether the role of MCH/MCHR1 in the LC in depression-like behaviors is associated with the regulation of norepinephrine.

**Method:**

Chronic unpredictable stress (CUS) and an acute intra-LC microinjection of MCH induced depression-like behaviors in rats. The MCHR1 antagonist SNAP-94847 was also microinjected in the LC in rats that were suffering CUS or treated with MCH. The sucrose preference, forced swim, and locomotor tests were used for behavioral evaluation. Immunofluorescence staining, enzyme-linked immunosorbent assay, western blot, and high-performance liquid chromatography with electrochemical detection were used to explore the mechanism of MCH/MCHR1 in the regulation of depression-like behaviors.

**Results:**

CUS induced an abnormal elevation of MCH levels and downregulated MCHR1 in the LC, which was highly correlated with the formation of depression-like behaviors. SNAP-94847 exerted antidepressant effects in CUS-exposed rats by normalizing tyrosine hydroxylase, dopamine β hydroxylase, and norepinephrine in the LC. An acute microinjection of MCH induced depression-like behaviors through its action on MCHR1. MCHR1 antagonism in the LC significantly reversed the MCH-induced downregulation of norepinephrine production by normalizing MCHR1-medicated cAMP-PKA signaling.

**Conclusions:**

Our study confirmed that the MCH/MCHR1 system in the LC may be involved in depression-like behaviors by downregulating norepinephrine production. These results improve our understanding of the pathogenesis of depression that is related to the MCH/MCHR1 system in the LC.

**Significance Statement**

Our previous work found that the melanin-concentrating hormone (MCH) system in the locus coeruleus (LC) may be involved in regulating depression-like behaviors. The present study further verified that MCH is closely related to the formation of depression-like behaviors in rats suffered chronic unpredictable stress (CUS). We found that CUS and an intra-LC microinjection of MCH reduced levels of norepinephrine, its synthetase tyrosine hydroxylase, and dopamine β hydroxylase, leading to the formation of depression. Direct inhibition of the MCHR1 receptor in the LC mitigated depression-like behaviors by restoring levels of norepinephrine and its synthetase in the LC. These results provide novel insights into the pathogenesis of depression and the involvement of MCH/MCHR1 in the LC.

## INTRODUCTION

Melanin-concentrating hormone (MCH) is a peptidergic neuromodulator primarily synthesized by neurons in the lateral hypothalamus (LH) (Bittencourt et al., 1992; Arrigoni et al., 2019). MCH exerts its effects through the G protein-coupled receptors MCH receptor 1 (MCHR1) and MCHR2. In rodents, only MCHR1 is functional (Torterolo et al., 2015). MCHR1 is reported to be widely expressed in the mammalian central nervous system (Saito et al., 2001), including the prefrontal cortex, hippocampus, dorsal raphe nucleus, and locus coeruleus (LC), among other regions. The extensive distribution of MCH and MCHR1 suggests that the MCHergic system may be involved in the regulation of various physiological functions, particularly mood and stress responses, by influencing neuronal activity and neurotransmitter function in the central nervous system (Devera et al., 2015).

Multiple studies have suggested that MCH/MCHR1 modulates depression-like behavior (Borowsky et al., 2002; Chaki et al., 2005; David et al., 2007). Chronic restraint stress upregulated MCH levels in the LH, hippocampus, and basolateral amygdala, brain regions important for emotional regulation (Kim and Han, 2016). Intraperitoneal administration of the MCHR1 antagonist SNAP-94847 was reported to significantly reverse anxiety- and depression-like behaviors induced by chronic stress (Smith et al., 2009; Kim et al., 2015). Genetic breed low responder mouse with a sign of depression-like behaviors exhibited a significant increase in hypothalamic pro-MCH mRNA levels and decrease in hippocampal MCHR1 mRNA levels (García-Fuster et al., 2012). Another study found that hippocampal MCHR1 gene expression significantly increased in a mouse model of chronic mild stress-induced depression (Roy et al., 2007). These results suggest that repeated stress induces abnormalities of MCH/MCHR1 in the central nervous system, which may be closely related to the progression of depression-like behaviors.

The LC is a stress-sensitive brain region in the pons, with noradrenergic neurons that project throughout the CNS, including the medial prefrontal cortex (mPFC), which plays an important role in regulation of the stress response, depression-like behaviors, and sleep (Benarroch, 2018). The LC also receives MCHergic projections from the LH (Del Cid-Pellitero and Jones, 2012; Yoon and Lee, 2013). In our previous study, we verified the existence MCHergic neurofibers in the LC and found that an acute microinjection of MCH in the LC elicited depression-like effects. This behavioral effect may be mediated by MCHR1, in which the MCHR1 antagonist SNAP-94847 blocked depression-like behaviors that was induced by MCH (Ye et al., 2018). Our findings suggest that MCH/MCHR1 in the LC may be closely related to the onset and progression of depression. However, the exact mechanism by which MCH/MCHR1 in the LC is involved in modulating depression-like behaviors remains unclear. Further studies are needed to elucidate the mechanisms of chronic stress- and MCH-induced neuronal dysfunction in the LC to better understand the role of LC MCH/MCHR1 in depression-like behaviors.

Chronic unpredictable stress (CUS) is a classic and widely used method for inducing depression-like behaviors in rats with high predictability and construct validity (Zeldetz et al., 2018). The present study used a CUS procedure to investigate the correlation between MCH/MCHR1 in the LC and the formation of depression and whether the mechanism involves the regulation of norepinephrine (NE) in the LC. We also verified the correlation between the antidepressant mechanism of MCHR1 antagonism in the LC and NE regulation in the rats that exhibited depression-like behavior induced by microinjection of MCH in the LC.

## MATERIALS AND METHODS

### Animals

Male Sprague-Dawley rats (250–300 g, Grade I, purchased from the Animal Center of Peking University, Beijing, China) were individually housed in plastic cages and maintained under an artificial 12-hour-light/-dark cycle (lights on 9:00 pm to 9:00 am) at 25°C ± 1°C and 50% ± 10% humidity. The rats had ad libitum access to food and water, except during specific CUS stressors. All experiments were in accordance with the National Research Council’s Guide for the Care and Use of Laboratory Animals and approved by the Peking University Animal Care and Use Committee (permission NO. LA2020279).

### Drugs

MCH was purchased from Phoenix Pharmaceutical (Burlingame, CA, USA) and dissolved in saline. The MCHR1 antagonist SNAP-94847 was obtained from Tocris (catalog NO. 3347; Minneapolis, MN, USA) and dissolved in 30% dimethyl sulfoxide. We did not observe any adverse effects of dimethyl sulfoxide in rats at this concentration, which is consistent with our previous report (Ye et al., 2018).

### Experimental Design

**Figure 1.**
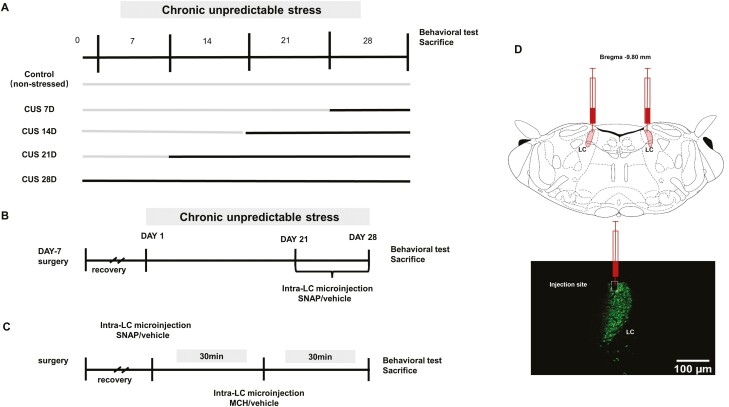
Time course of the experiment and histological identification of the microinjection sites. (A–C) The time courses of the first, second, and third experiments. (D) Photomicrograph scan of a coronal section illustrating the localization of locus coeruleus (LC) and injection site.

In the first experiment, rats were exposed to 7, 14, 21, and 28 days of CUS. Behavioral experiments included the sucrose preference test (SPT), locomotor test, and the forced swim test (FST), which were performed after the CUS procedure (Figure 1A). All animals were randomly divided into 2 separate cohorts after the behavioral tests. One cohort was killed, and LH and LC tissues were harvested for western-blot and enzyme-linked immunosorbent assay (ELISA) analyses. The second cohort underwent perfusion and fixation for further immunofluorescence staining.

In the second experiment, rats underwent surgery to implant guide cannulas for the LC microinjections. After 7 days of recovery from surgery, the rats underwent 28 days of CUS, and SNAP-94847 (6 μg in 0.2 μL/site) or 30% dimethyl sulfoxide was bilaterally microinjected in the LC daily starting on day 22 for 7 consecutive days. The behavioral tests were performed 60 minutes after the last microinjection (Figure 1B). The dose of MCH was based on our previous study (Ye et al., 2018). At least 7 days elapsed between experiments. The rats were killed after the behavioral tests, and LC and mPFC tissues were harvested for further analysis. Data from cannula misplacements, cannula blockage, and extensive damage of LC have been excluded.

In the third experiment, rats underwent surgery to implant guide cannulas for the LC microinjections. After 7 days of recovery from surgery, SNAP-94847 (6 μg in 0.2 μL/site) or 30% dimethyl sulfoxide was bilaterally microinjected in the LC 30 minutes before the MCH microinjection. MCH (100 ng in 0.2 μL/site) or saline was bilaterally microinjected in the LC 30 minutes before the behavioral tests (Figure 1C). The behavioral tests were performed 60 minutes after the last microinjection. The rats were killed after the behavioral tests, and LC and mPFC tissues were harvested for further analysis. Data from cannula misplacements, cannula blockage and extensive damage of LC were excluded.

### Cannula Implantation Surgery and Drug Infusion in the LC

Surgical procedures were performed as previously described (Ye et al., 2018). Briefly, the rats were i.p. anesthetized with 10% chloral hydrate and positioned in a stereotaxic apparatus. A double-guide cannula (26-gauge, C/C dist. 2.4 mm, RWD Life Science, Shenzhen, China) was implanted with the tip 1 mm above the LC at the following coordinates (distance from lambda): anterior/posterior, −9.8 mm; lateral, ± 1.2 mm; dorsal/ventral, −6.0 mm below the brain surface (15° inclination of vertical stereotaxic bar). The cannula was secured to the skull with 4 stainless-steel screws and dental acrylic. After surgery, the rats were injected with penicillin for 3 days and allowed to recover for 7 days before the following experiments.

The drug infusions were performed with a Hamilton syringe connected to a 33-gauge injection cannula (RWD Life Science) in the home cage. MCH/SNAP or vehicle was microinjected through the injection cannula that extended 1 mm beyond the guide cannula in a 0.2-μL volume for 2 minutes. The injection cannula was unmoved for another 2 minutes for the drug to completely diffuse from the tip. The injection placements were verified by immunofluorescence staining or unaided eye and are shown in Figures 1D, 4A, and 5A. All data presented in this study were derived from animals whose injection sites were within the LC.

### CUS Procedure

CUS is a commonly used method for inducing depression-like behaviors in which animals undergo a random sequence of stressors. The CUS procedure was slightly modified from previous studies (Pastor-Ciurana et al., 2014; Sun et al., 2016). The rats were exposed to 12 different stressors (2 stressors per day), including 4 types of strong stressors (footshock, tail pinch, body restriction, and ice-water bath) and 8 types of mild stressors (bright light stimulation, water or food deprivation, white noise, cage shaking, wet cage, crowded housing, tilted cage, and inverse light/dark cycle) (supplementary Table 1 for CUS procedure). To avoid predictability, the rats were exposed to these stressors at different times each day. Rats in the control group were left undisturbed in their home cages while rats in the CUS group were exposed to various stress conditions.

### Behavioral Tests

On the day of the behavioral tests, the SPT, locomotor test, and FST were conducted sequentially. To minimize confounding factors, the animals were allowed to rest for at least 1 hour between each experiment. Furthermore, the rats were killed at least 1 hour after completing the behavioral tests to mitigate neurochemical changes induced by acute stress from the FST.

### Sucrose Preference Test

The SPT was used to determine anhedonia-like behavior, which is considered a core symptom of clinical depression (Liu MY et al., 2018). In the present study, the rats were habituated to drinking from 2 bottles for 48 hours. One bottle was filled with water, and the other bottle was filled with 1% sucrose solution. After training, the rats were water deprived for 24 hours before the SPT. In the first and second experiments, water deprivation was combined with CUS and performed on the last day of procedure. On the test day, the water and 1% sucrose bottles were placed in the rat’s home cages, and rats were allowed to drink freely from both bottles for 1 hour. Water and sucrose consumption were measured by comparing the weight difference of the bottles before and after the test. Sucrose preference was calculated according to the following formula: sucrose preference = sucrose intake (g)/ total intake (sucrose intake [g] + water intake [g]) × 100%.

### Locomotor Test

Locomotor activity was measured by the locomotor test, which was performed as previously described (Ye et al., 2018). The rats were placed in a Plexiglas chamber (40 cm × 40 cm × 65 cm), and behavior was recorded by an automated video tracking system (DigBehv-LM4, Shanghai Jiliang Software Technology, Shanghai, China). The video files were later analyzed using DigBehv analysis software. Locomotor activity is expressed as the total distance traveled in 10 minutes.

### Forced Swim Test

The FST was performed according to a modified version of the paradigm (Yankelevitch-Yahav et al., 2015). On the pretest day, each rat was individually placed for 15 minutes into a 25-cm-diameter × 60-cm-high Plexiglas cylinder filled with 23°C to 25°C water to a depth of 40 cm. On the test day, the rat was placed into the same cylinder again and recorded for 5 minutes. Behavior was recorded by 2 video cameras (1 on top and 1 on the side). After the experiment, the rat was removed from the water, dried with a towel, and returned to its home cage. The videotapes were analyzed by a researcher who was blind to each rat’s treatment condition. Immobility was defined as the minimum movement that was necessary to keep the rat’s head above the water.

### Western-Blot Analysis

Rats were decapitated after the behavioral tests. Immediately after decapitation, the brains were quickly removed to a prechilled brain matrix. Bilateral punches (2-mm diameter) of the LC (from bregma, −9.16 to approximately −10.52 mm) and surrounding tissue were separated with a hypodermic needle guided by the Paxinos and Watson rat brain atlas (Paxinos and Watson, 2005). The process was performed on ice. LC tissues were homogenized in RIPA lysis buffer supplemented with protease and phosphatase inhibitors. The samples were centrifuged at 12 000 rpm for 15 minutes at 4°C, and the supernatants were collected. Equal amounts of protein (25 μg) were resolved by 10% sodium dodecyl sulfate-polyacrylamide gel electrophoresis and transferred to polyvinylidene difluoride membranes (IPVH00010, Merck Millipore, Shanghai, China). The blots were blocked with 5% skim milk for 1 hour at room temperature followed by incubation overnight at 4°C with the appropriate primary antibodies. The following primary antibodies were used: anti-MCHR1 (1:200; sc-100327, Santa Cruz, Shanghai, China), anti-tyrosine hydroxylase (TH, 1:1000; #58844, Cell Signaling Technology, Shanghai, China), anti-dopamine β-hydroxylase (DBH, 1:1000; ab209487, Abcam, Shanghai, China), anti-protein kinase A (PKA, 1:1000; #4782s, Cell Signaling Technology), anti-phospho-PKA (p-PKA, 1:1000; #5661s, Cell Signaling Technology), and anti-β-actin (1:5000; AC038, Abclonal, Wuhan, China). The blots were then washed with Tris buffered saline with Tween-20 3 times before incubation with goat anti-rabbit (1:2000; AS014, Abclonal) or anti-mouse (1:2000; AS003, Abclonal) secondary antibodies for 2 hours at room temperature. Finally, an ECL Enhanced kit (catalog No. RM00021, Abclonal) was used for detection enhancement, and blots were visualized using ImageJ software. All the results were normalized to the protein expression level of β-actin. CUS stress or drug treatments alone did not impact β-actin levels (supplementary Fig. 1).

### Immunofluorescence Staining

The rats were perfused with 4% paraformaldehyde in phosphate-buffered saline (PBS; pH 7.4). Whole brains were removed and postfixed in the 4% paraformaldehyde for 24 hours and then dehydrated in 30% sucrose for cryoprotection. The brains were rapidly frozen on liquid n-hexane that was cooled with a mixture of solid carbon dioxide and ethanol. Coronal cryostat sections (20 μm) that encompassed the LH (bregma −1.6 to approximately −2.8 mm) or LC (−9.16 to approximately −10.52 mm) were cut using a freezing microtome (Leica CM1850, Leica Microsystems UK, Milton Keynes, UK) based on a rat brain atlas. The antigen retrieval of the sections was conducted in citrate buffer (pH 6.0) via microwave. After the sections were returned to room temperature naturally, they were immersed in PBS containing 5% nonspecific donkey serum and 0.3% Triton X-100 for 30 minutes. The sections were incubated in the primary antibodies for MCH (1:200; H-070-47, Phoenix pharmaceuticals, LH slides) or TH (1:500; sc-25269, Santa Cruz, LC slides) diluted in PBS containing 1.5% donkey nonspecific serum for 16 hours at 4°C. After washing with PBS 3 times for 5 minutes, sections were incubated with fluorophore-conjugated secondary antibodies (1:2000; ab150073, Donkey anti-rabbit IgG Alexa Fluor 488, Abcam; 1:500; AS077, Goat anti mouse IgG Alexa Fluor 594, Abclonal) for 2 hours at room temperature. Finally, the sections were mounted with fluorescent mounting medium with 4ʹ,6-diamidino-2-phenylindole. Single images of the LH for each rat were scanned using a Nikon DS-Ri2 microscope camera (Nikon, Tokyo, Japan) and processed by Image J software. The figures were merged with 4ʹ,6-diamidino-2-phenylindole (blue) staining to clearly display MCH-positive neurons (green). All images of each section were acquired at 20× magnification. The number of MCH-positive neurons in each mm^2^ LH area was evaluated according to our common protocol (Cui et al., 2020). At least 3 serial sections of the LH were used to analyze the data for each rat. There were n = 5 animals per group.

### Enzyme‑linked Immunosorbent Assay

LC and LH tissues were harvested and homogenized in RIPA buffer with protease inhibitors, and supernatants were collected. Commercial ELISA kits (EK-070-47, Phoenix Pharmaceuticals) were performed according to the manufacturer’s instructions. Briefly, sample or standard along with the binding protein and biotinylated peptide were added to each well and incubated for 120 minutes at room temperature. Then horseradish peroxidase conjugated streptavidin was added to each well and incubated for 60 minutes at room temperature. Thereafter, substrate solution was added and incubated for 60 minutes at room temperature, followed by the addition of 2 N HCL to each well to stop the reaction. Finally, the immune plate was loaded onto a Multiskan MK3 microplate reader (Thermo, USA). Absorbance (optical density) was read at 450 nm.

LC tissue was harvested and homogenized in 0.1 M HCl, and supernatants were collected. Cyclic adenosine monophosphate (cAMP) levels in clarified lysates were measured using the direct cAMP ELISA kit (ADI-900-066, Enzo Life Sciences, Farmingdale, NY, US) according to the manufacturer’s protocol. The neutralizing reagent, standard or sample, conjugate, and cAMP antibody were added to each well and incubated for 120 minutes at room temperature. After washing, substrate solution was added to each well and incubated for 60 minutes at room temperature. Finally, stop solution was added to each well to stop the reaction. Absorbance (optical density) was read at 405 nm using a Multiskan MK3 microplate reader.

The concentrations of MCH and cAMP in each sample were normalized to the corresponding tissue weight.

### High-Performance Liquid Chromatography With Electrochemical Detection (HPLC-ECD)

The LC and mPFC tissue were dissected and extracted with 0.2 M perchloric acid by ultrasonic homogenization. Details of the neurotransmitter analysis procedure were previously described (Huang et al., 2016). HPLC-ECD was used to determine NE levels under the following conditions: flow rate (0.60 mL/min), temperature (40°C), column (Shiseido Capcell Pak C18 MG F90816 column; 3.0-mm inner diameter, 75-mm length, 3-μm pore size), injection volume (20 μL partial loop), mobile phase (0.1 M NaH_2_PO_4_, 0.85 mM OSA, 0.05 mM EDTA-2Na, 11% CH_3_OH, pH 3.25 with H_3_PO_4_), detector, and conditions (analytical cell: 5011A, E1 = −175 mV, E2 = +200 mV; guard cell: 5020, EGC = +250 mV).

### Statistical Analysis

GraphPad Prism 9 software (GraphPad Software Inc., San Diego, CA, USA) was used for all statistical analyses. Data are expressed as the mean ±  SEM. Group comparisons were performed by 1-way ANOVA or 2-way ANOVA followed by the Newman‒Keuls post hoc test. For 2-way ANOVA, the procedure (control or CUS) or (vehicle or MCH) and the treatment (vehicle or SNAP-94847) were taken as between-group factor. In all analyses, differences were considered statistically significant at *P* < .05. The correlation between immobility time or sucrose preference and MCH levels was assessed using Pearson correlation analysis. Correlation analysis was performed on pooled data for LH and LC from both controls and CUS rats at all groups.

## Results

### Correlation Between CUS-Induced Depression-Like Behaviors and the MCHergic System

**Figure 2.**
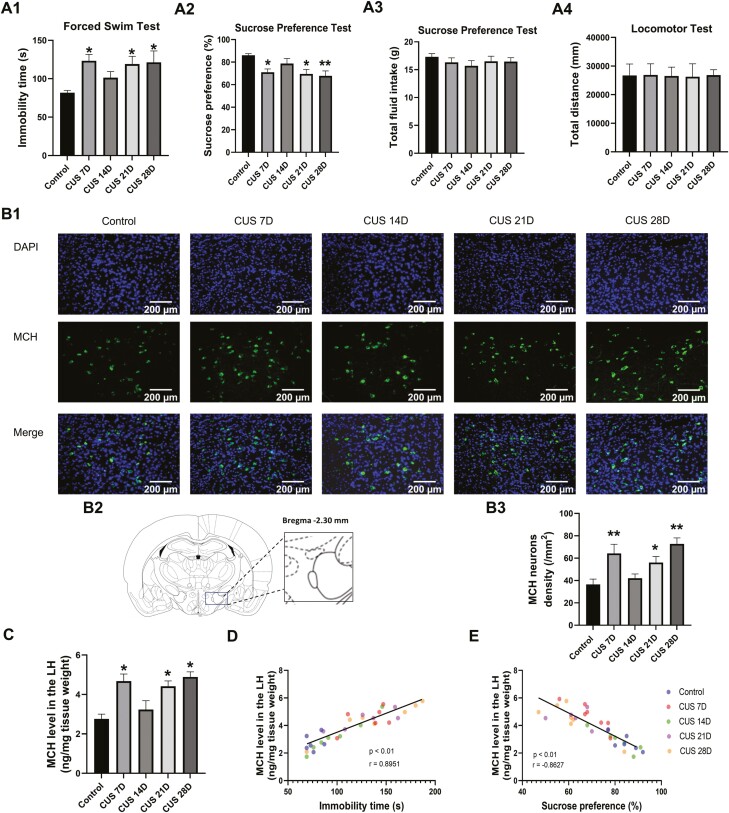
Correlation between melanin-concentrating hormone (MCH) levels in the lateral hypothalamus (LH) and depression-like behavior. (A1-A3) Chronic unpredictable stress-induced depression-like behavior. Immobility time in the forced swim test (FST), sucrose preference, total fluid intake in the sucrose preference test (SPT), and total distance traveled in the locomotor test were evaluated (n = 10/group). (B1-B2) Representative photographs of immunostained sections showing MCH+ neurons in the LH (B1) and quantification of the MCH+ cell ratio (B2; n = 5/group). (C) MCH levels in the LH (n1, n2, n3, n4, n5 = 6, 7, 7, 6, 6). (D) Correlation between MCH levels in the LH and immobility in the FST (r = 0.8951, *P* < .01). (E) Correlation between MCH levels in the LH and sucrose preference in the SPT (r = −0.8627, *P* < .01). The data are expressed as the mean ± SEM. *P < .05, **P < .01 vs control group (1-way ANOVA followed by Newman-Keuls post hoc test).

Different durations of CUS were tested to investigate the correlation between depression-like behaviors and changes in MCH levels in the LH. Immobility time in the FST, sucrose preference in the SPT, and total distance traveled in the locomotor test were determined. Over the different time courses of CUS, clear behavioral changes were observed in the FST (F_(4,45)_ = 4.234, *P* < .01; Figure 2A1) and SPT (F_(4,45)_ = 4.542, *P* < .01; Figure 2A2). The post hoc analysis revealed that the CUS 7D, CUS 21D, and CUS 28D groups but not the CUS 14D group exhibited a significant increase in immobility time (Figure 2A1) and a significant decrease in sucrose preference (Figure 2A2) compared with the control (non-CUS) group, without significant effect on total fluid intake (F_(4,45)_ = 0.5251, *P* > .05; Figure 2A3). Moreover, the locomotor activity was evaluated by the locomotor test, and no significant alterations of the total distance traveled were observed (F_(4,45)_ = 0.01372, *P* > .05; Figure 2A4). These results indicate that locomotor activity of the rats was not affected by CUS and further imply that the changes in immobility time that we observed in the FST were not related with locomotor ability of rats.

MCH levels in the LH were tested using both immunofluorescence staining and ELISA. The immunofluorescence staining of MCH was used to quantify the density of MCH-positive neurons in the LH (Figure 2B1). The density of MCH-positive neurons in the LH showed a U-shaped trend with different CUS durations (F_(4,20)_ = 7.144, *P* < .01; Figure 2B2). The post hoc analysis revealed that after 7, 21, and 28 days of CUS, MCH levels in the LH significantly increased compared with the control (non-CUS) group. CUS for 14 days did not affect MCH levels. The ELISA results were the same as above. MCH levels in the LH also exhibited a U-shaped trend with different CUS times (F_(4,27)_ = 7.346, *P* < .01; Figure 2C). The post hoc analysis revealed that the CUS 7D, CUS 21D, and CUS 28D groups but not the CUS 14D group exhibited a significant increase in MCH levels in the LH.

To further evaluate correlations between MCH levels and depression-like behaviors, Pearson correlation analysis was performed to illustrate possible relationships between immobility time in the FST (an indicator of “despair”) and MCH levels in the LH in all experimental groups. The results showed that immobility time positively correlated with MCH levels in the LH (r = 0.8951, *P* < .01; Figure 2D). We also evaluated correlations between MCH levels and sucrose preference in the SPT (an indicator of “anhedonia”) and found a negative correlation between sucrose preference and MCH levels in the LH (r = −0.8627, *P* < .01; Figure 2E). These results indicated a strong linear relationship between depression-like behaviors and MCH levels in the LH.

**Figure 3.**
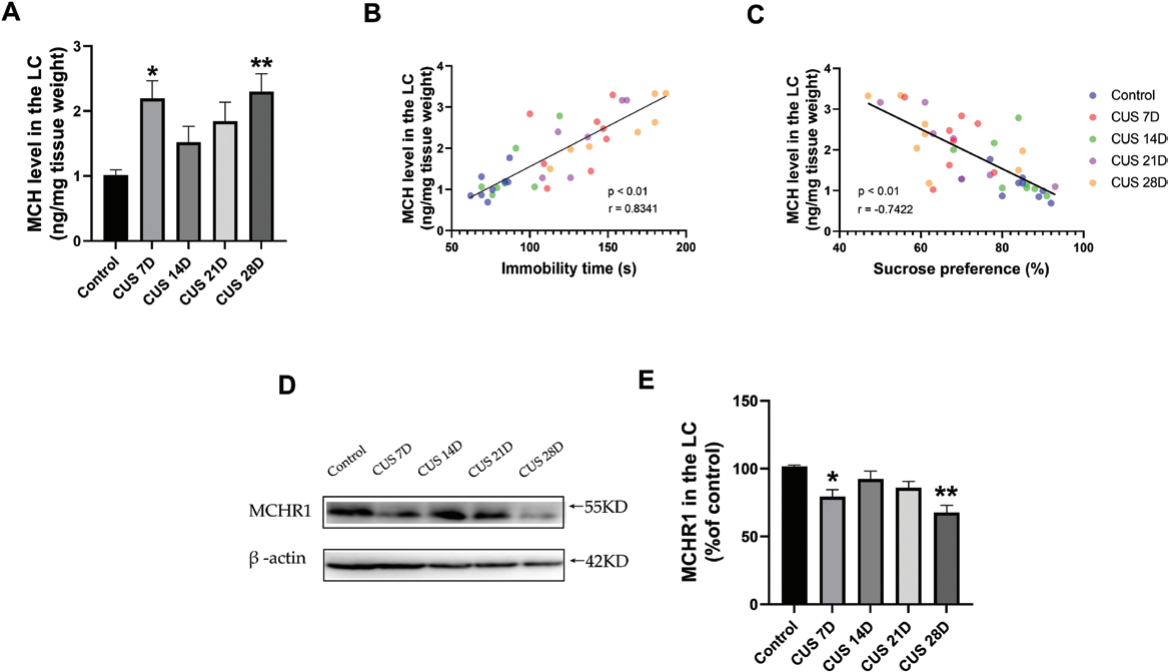
Correlation between melanin-concentrating hormone (MCH) or MCH receptor 1 (MCHR1) level in the locus coeruleus (LC) and depression-like behavior. (A) MCH levels in the LC (n1, n2, n3, n4, n5 = 7, 8, 8, 7, 8). (B) Correlation between MCH levels in the LC and immobility time in the forced swim test (FST) (r = 0.8341, *P* < .01). (C) Correlation between MCH levels in the LC and sucrose preference in the sucrose preference test (SPT) (r = −0.7422, *P* < .01). (D) Western-blot images of MCHR1 and β-actin. (E) Western-blot analysis of MCHR1 at different chronic unpredictable stress (CUS) times (n = 8/group). The data are expressed as the mean ± SEM. *P < .05, **P < .01 vs control group (1-way ANOVA followed by Newman-Keuls post hoc test).

We further determined changes in MCH levels in the LC, and the results showed a similar trend as in the LH (F _(4,33)_ = 4.282, *P* < .01; Figure 3A). The post hoc analysis revealed that only 7 and 28 days of CUS significantly increased MCH levels in the LC, and 21 days of CUS slightly but nonsignificantly elevated MCH levels. Fourteen days of CUS did not affect MCH levels in the LC (Figure 3A). The correlation between MCH levels in the LC and depression-like behaviors was determined by Pearson correlation analysis. The results indicated that immobility time positively correlated (r = 0.8341, *P* < .01; Figure 3B), and sucrose preference negatively correlated (r = −0.7422, *P* < .01; Figure 3C) with MCH levels in the LC. These results indicated a strong linear relationship between depression-like behaviors and MCH levels in the LC.

To explore whether MCH acts through MCHR1 to induce depression-like behaviors, we investigated the expression of MCHR1 in the LC (Figure 3D). Interestingly, we found that MCHR1 expression significantly decreased after 7 and 28 days of CUS. There was a slight but nonsignificant reduction after 14 or 21 days of CUS (F_(4,35)_ = 7.402, *P* < .01; Figure 3E). Whereas MCH levels increased, MCHR1 expression significantly decreased, which was exactly the time when depression-like behaviors occurred.

### Intra-LC Microinjection of SNAP-94847 Reversed CUS-Induced Depression-Like Behaviors

**Figure 4.**
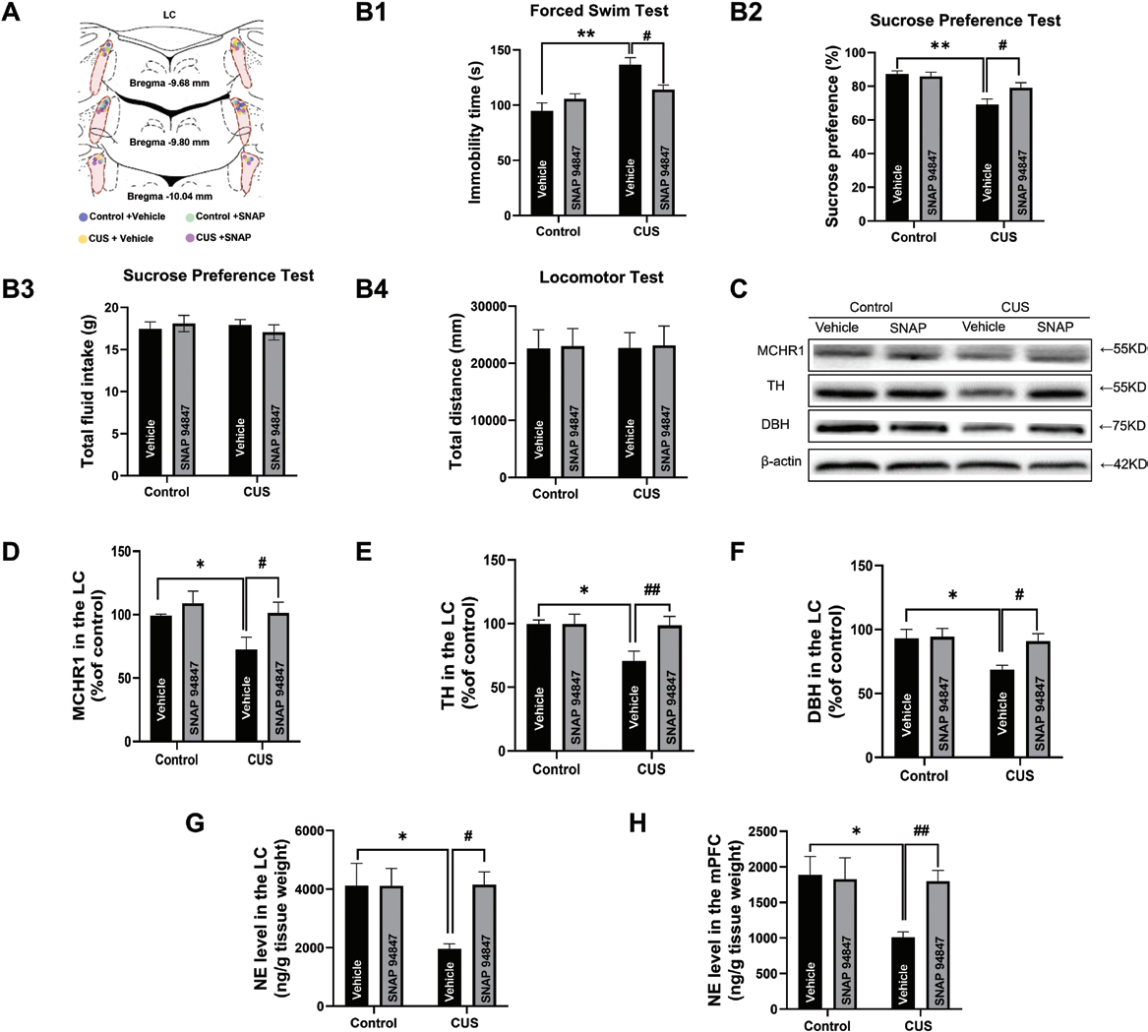
Intra-locus coeruleus (LC) microinjection of SNAP-94847 reversed chronic unpredictable stress (CUS)-induced depression-like behavior and restored norepinephrine (NE) levels by increasing tyrosine hydroxylase (TH) and dopamine β-hydroxylase (DBH) expression. (A) Schematic diagram of LC microinjection site. (B1–B4) The melanin-concentrating hormone receptor 1 (MCHR1) antagonist SNAP-94847 significantly reversed depression-like behavior that was induced by CUS. Immobility time in the forced swim test (FST), sucrose preference total fluid intake in the sucrose preference test (SPT), and total distance traveled in the locomotor test were evaluated (n1, n2, n3, n4 = 6, 6, 7, 7). (C) Western-blot images of MCHR1, TH, DBH, and β-actin. (D–F) Western-blot analysis of MCHR1, TH, and DBH expression (n = 6/group). (G–H) NE levels in the LC (n1, n2, n3, n4 = 9, 10, 10, 10) and medial prefrontal cortex (mPFC) (n1, n2, n3, n4 = 8, 10, 9, 10). The data are expressed as the mean ± SEM. *P < .05, **P < .01 vs control-vehicle treated group; ^#^*P* < .05 and ^##^*P* < .01 vs CUS-vehicle treated group (2-way ANOVA followed by Newman-Keuls post hoc test).

To elucidate the involvement of LC MCH/MCHR1 in behavioral alterations induced by CUS, the MCHR1 antagonist SNAP-94847 was microinjected in the LC starting on day 21 for 7 consecutive days during the CUS time course. CUS significantly increased immobility time in the FST (F_(1,22)_ = 18.59, *P* < .01; Figure 4B1) and decreased sucrose preference in the SPT (F_(1,22)_ = 19.61, *P* < .01; Figure 4B2) without significant effect on total fluid intake (F_(1,22)_ = 0.8394, *P* >.05; Figure 4B3). The microinjection of SNAP-94847 in the LC for 7 days reversed CUS-induced depression-like behaviors by reducing immobility time in the FST (F_(1,22)_ = 8.329, *P* < .05; Figure 4B1) and recovering sucrose preference in the SPT (F_(1,22)_ = 4.189, *P* < .05; Figure 4B2). These anti-depressant effects of SNAP-94847 were not significant when it was microinjected outside the LC (supplementary Fig. 2). There were no significant changes in the total distance traveled in the locomotor test (Figure 4B4).

The western-blot analysis of MCHR1 expression in the LC was conducted to verify the antidepressant effect of its antagonism on CUS-induced depression-like behaviors (Figure 4C). We found that CUS significantly decreased MCHR1 expression levels in the LC (F_(1,20)_ = 4.698, *P* < .05), and the intra-LC microinjection of SNAP-94847 restored MCHR1 expression (F_(1,20)_ = 5.936, *P* < .05; Figure 4D).

### Intra-LC Microinjection of SNAP-94847 Recovered CUS-Induced NE Downregulation

The western-blot analysis of tyrosine hydroxylase (TH) and dopamine β-hydroxylase (DBH) (Figure 4C) revealed that CUS significantly reduced TH (F_(1,20)_ = 4.362, *P* < .05) and DBH (F_(1,20)_ = 5.405, *P* < .05) expression levels. The intra-LC microinjection of SNAP-94847 reversed the decrease in both TH (F_(1,20)_ = 5.170, *P* < .05) and DBH (F_(1,20)_ = 4.047, *P* < .05) expression, suggesting the recovery of NE synthesis (Figure 4E–F). HPLC-ECD was performed to detect NE levels in the LC (Figure 4G). The results showed that CUS significantly reduced NE levels in the LC (F_(1,35)_ = 4.258, *P* < .05), which was recovered by the intra-LC microinjection of SNAP-94847 (F_(1,35)_ = 4.322, *P* < .05). The primary source of NE in the mPFC includes afferents from the LC. To investigate the LC-mPFC noradrenergic projection, we also examined NE levels in the mPFC (Figure 4H). The results showed that CUS significantly reduced NE levels (F_(1,33)_ = 4.906, *P < *.05), and the intra-LC microinjection of SNAP-94847 reversed the decrease in NE levels (F_(1,33)_ = 5.553, *P < *.01) in the mPFC, indicating restoration of the LC-mPFC noradrenergic projection.

### Intra-LC Microinjection of SNAP-94847 Blocked MCH-Induced Depression-Like Behaviors and cAMP-PKA Signaling

**Figure 5.**
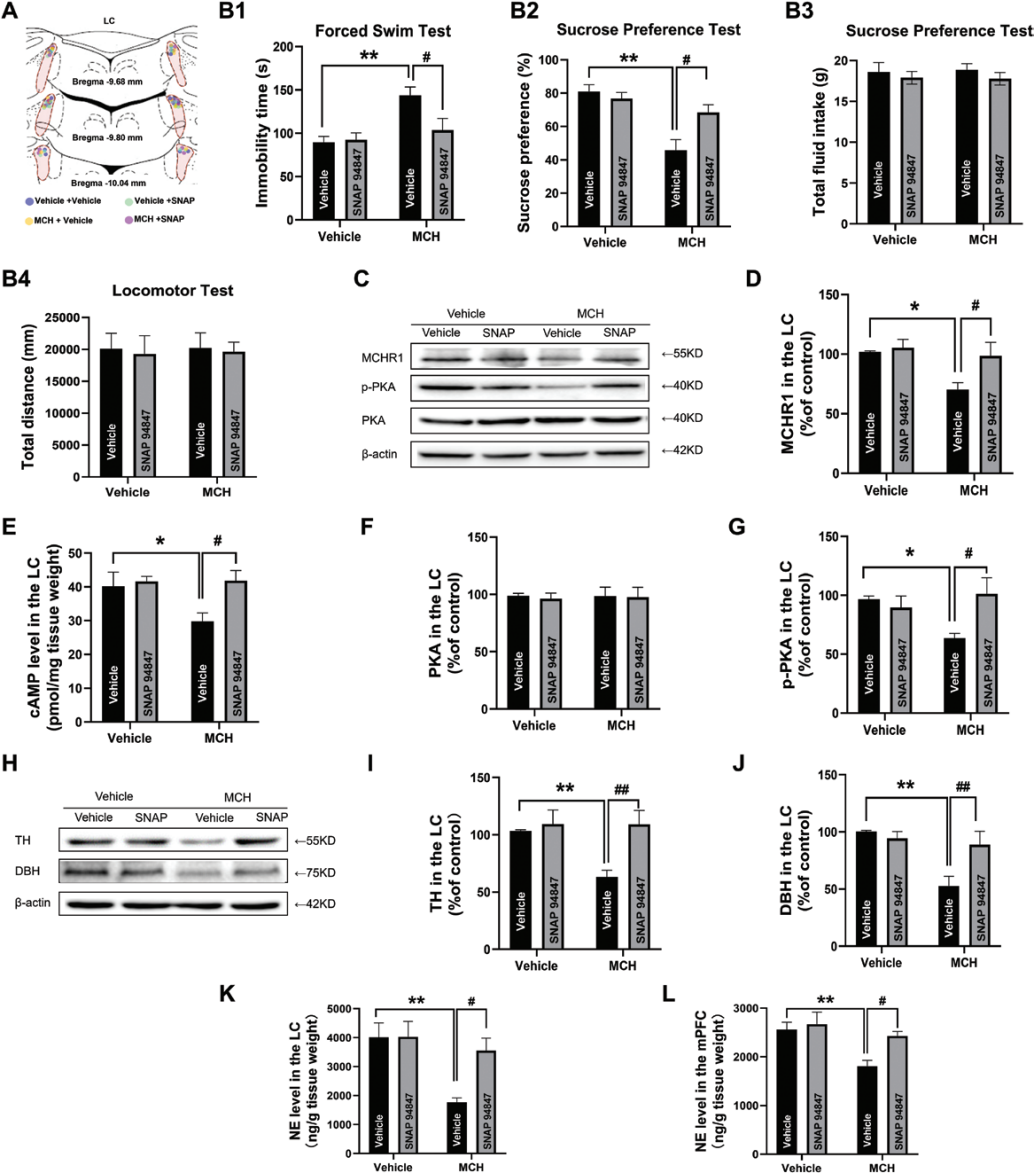
Pretreatment with SNAP-94847 in the locus coeruleus (LC) recovered the melanin-concentrating hormone (MCH)-induced cell signaling and inhibition on norepinephrine (NE) production. (A) Schematic diagram of LC microinjection site. (B1–B4) The intra-LC microinjection of MCH induced depression-like behavior, which was reversed by pretreatment with the MCHR1 antagonist SNAP-94847. Immobility time in the forced swim test (FST), sucrose preference total fluid intake in the sucrose preference test (SPT), and total distance traveled in the locomotor test were evaluated (n1, n2, n3, n4 = 9, 10, 9, 10). (C) Western-blot images of melanin-concentrating hormone receptor 1 (MCHR1), protein kinase A (PKA), phospho-PKA (p-PKA), and β-actin. (D–G) MCHR1 expression, cyclic adenosine monophosphate (cAMP) level, PKA expression, and p-PKA expression (n = 6/group). (H) Western-blot images of tyrosine hydroxylase (TH), dopamine β-hydroxylase (DBH), and β-actin. (I–J) TH and DBH expression (n = 6/group). (K–L) NE levels in the LC and medial prefrontal cortex (mPFC) (n = 12/group). The data are expressed as the mean ± SEM. *P < .05, **P < .01 vs vehicle-vehicle treated group; ^#^*P* < .05 and ^##^*P* < .01 vs MCH treated-vehicle group (2-way ANOVA followed by Newman-Keuls post hoc test).

The intra-LC microinjection of MCH increased immobility time in the FST (F_(1,34)_ = 10.53, *P* < .01; Figure 5B1) and decreased sucrose preference in the SPT (F_(1,34)_ = 20.46, *P* < .01; Figure 5B2) without significant effect on total fluid intake (F_(1,34) = _0.04787, *P* >.05; Figure 5B3). Pretreatment with SNAP-94847 in the LC blocked MCH-induced depression-like behaviors in the FST (F_(1,34)_ = 4.550, *P < *.05) and SPT (F_(1,34)_ = 7.780, *P < *.05). No significant alterations of the total distance traveled were found in the locomotor test (Figure 5B4), in agreement with previous reports (Ye et al., 2018). However, when MCH was microinjected outside the LC, depression-like behaviors detected by the FST and SPT were not significant. Meanwhile, no significant behavioral changes observed with SNAP-94847 pretreatment rats with injection site outside the LC (supplementary Fig. 3).

To identify downstream mechanisms involved in MCH-induced depression-like behaviors in the LC, MCHR1 expression, intracellular cAMP levels, and protein kinase A (PKA) and phosphorylated PKA (p-PKA) expression were investigated. MCHR1 expression significantly decreased after the acute intra-LC microinjection of MCH (F_(1,20)_ = 7.104, *P* < .05; Figure 5C–D), and pretreatment with the MCHR1 antagonist SNAP-94847 significantly restored its expression level (F_(1,20)_ = 4.931, *P < *.05). The ELISA results showed that the acute intra-LC microinjection of MCH reduced intracellular cAMP levels (F_(1,20)_ = 5.310, *P < *.05; Figure 5E), which was also reversed by pretreatment with SNAP-94847 (F_(1,20)_ = 4.931, *P* < .05). There were no significant changes in PKA levels (Figure 5C, F), whereas p-PKA expression significantly decreased after the acute intra-LC microinjection of MCH (F_(1,20)_ = 4.497, *P < *.05; Figure 5B, G), and pretreatment with SNAP-94847 increased its expression level.

### Intra-LC Microinjection of SNAP-94847 Reversed the Inhibitory Effect of MCH on NE

The western-blot analysis of TH and DBH (Figure 5H) revealed that the intra-LC injection of MCH significantly reduced the expression of TH (F_(1,20)_ = 5.289, *P < *.01; Figure 5I) and DBH (F_(1,20)_ = 11.41, *P* < .01; Figure 5J). Pretreatment with SNAP-94847 reversed the decrease in both TH (F_(1,20)_ = 5.262, *P < *.05) and DBH (F_(1,20)_ = 7.217, *P* < .05) expression. These results suggest that the acute microinjection of MCH significantly downregulated NE synthesis. HPLC-ECD analysis was performed to detect NE levels in the LC (Figure 5K) and the mPFC (Figure 5L). The results showed that the MCH microinjection reduced NE levels in the LC (F_(1,44)_ = 10.01, *P* < .01) and the mPFC (F_(1,44)_ = 9.059, *P* < .01), and SNAP-94847 reversed this decrease in both LC (F_(1,44)_ = 4.261, *P* < .05) and mPFC (F_(1,44)_ = 4.862, *P* < .05), indicating the restoration of LC-mPFC noradrenergic transmission.

## Discussion

 Chronic exposure to stressful events is consistently considered as a high-risk factor for depression (Hammen, 2005; Antoniuk et al., 2019; Sousa et al., 2021). The CUS procedure is proposed to mimic socio-environmental stressors in inducing symptoms that share some characteristics of human depression, such as despair- and anhedonia-like behaviors. In this study, we assessed multiple dimensions of depression-like behavioral phenotypes using the SPT (anhedonia), FST (despair-like behavior), and locomotor test (motor activity). In the present study, we investigated changes in the MCH system with different durations of CUS, which provided an experimental basis for differences in depression-like behaviors during different stages of stress. Meanwhile, we further discovered that the activation of MCH in the LC by CUS and acute microinjection of MCH produced depression-like behaviors by downregulating NE production, which was reversed by the MCHR1 antagonist SNAP-94847.

Several lines of evidence indicate that MCH neural systems in the LC play a crucial role in regulating depression-like behaviors. Hypothalamic MCH innervation and MCHR1 expression in the LC were previously reported (Saito et al., 2001; Del Cid-Pellitero and Jones, 2012; Yoon and Lee, 2013). We observed clear behavioral changes that depended on the duration of CUS and found strong correlations between the formation of depression-like behaviors and MCH levels in both the LH and LC. Interestingly, changes in MCHR1 expression in the LC were opposite to MCH level with different stress durations. We hypothesized that when the organism is exposed to stress and the LH-LC MCHergic innervation is abnormally elevated and was followed by a downregulation in LC-MCHR1 expression. The change in MCH to MCHR1 ratio is a sign of compensatory response and might be associated with alterations in GPCR-mediated downstream signaling. When the organism adapts to the stress, as a consequence of homeostasis regulation, MCH/MCHR1 returns to normal, and the depression-like behaviors disappears. When the organism continues to be exposed to stress and exceeds the limit of adaptive adjustment, the LH-LC MCH innervation re-increased, accompanied by a decrease in MCHR1 expression and a recurrence of depression-like behaviors, such a strong correlation indicating chronic stress-induced abnormalities of the MCHergic system may be one of the important factors for depression-like behaviors. Meanwhile, we found that MCHR1 antagonism in the LC restored the CUS/MCH-induced downregulation of MCHR1 expression and ameliorated depression-like behaviors. Based on these results, we hypothesize that maintaining homeostasis of the MCH/MCHR1 system, especially MCHR1 in the LC, is essential for regulating depression-like behaviors.

The LC is the main source of NE in the central nervous system. Abnormal function of the noradrenergic system has been shown to be involved in the pathogenesis and treatment of depression (Betts et al., 2019). Although stress-induced noradrenergic alterations in the LC remain debatable, some believe that LC noradrenergic neuronal loss and decreased noradrenergic transmission is associated with the pathogenesis of depression (Weiss et al., 1996; Szot et al., 2016; Pan et al., 2021). Morphological studies have shown that the mPFC receives the highest density of NE varicosities relative to other brain regions (Agster et al., 2013). Several studies have also shown that noradrenergic functional connectivity between the LC and mPFC plays a key role in the regulation of depression-like behaviors and stress responses (Nutt, 2008; Marzo et al., 2014; Zitnik et al., 2015). A previous study suggested the possible inhibitory action of MCH on noradrenergic neurons in the LC, since the activity of noradrenergic neurons was significantly reduced or even stopped during rapid-eye-movement sleep, and MCH promoted the occurrence of rapid-eye-movement sleep (Monti et al., 2015). Additionally, an acute intra-LC microinjection of MCH significantly decreased extracellular NE levels in the prefrontal cortex, suggesting an inhibitory effect of MCH on noradrenergic neuronal activity (Urbanavicius et al., 2019). Noradrenergic function in the LC is reflected by levels of NE biosynthesis enzymes (Sabban et al., 2018). The present study showed that CUS-induced reductions of TH and DBH were restored by the chronic administration of an MCHR1 antagonist, suggesting that the CUS-induced dysfunction of NE synthesis in the LC may be associated with the MCHergic system. We also found that CUS or acute microinjection of MCH significantly decreased NE levels in both LC and mPFC, and MCHR1 antagonism restored NE levels and presumably recovered the LC-mPFC noradrenergic projection, and CUS-induced outcomes are consistent with earlier published findings (Yu et al., 2013; Ahmed et al., 2023). Our data demonstrated that MCH inhibited noradrenergic synthesis in the LC by acting on MCHR1, which might further diminish the NE projection to the mPFC as an inducement of the depression-like behaviors.

MCHR1 is a G protein-coupled receptor that can couple with G_i_ protein and inhibit cAMP-PKA signaling, resulting in lower adenylate cyclase activity, cAMP formation, and PKA levels and the inhibition of neuronal activation (Hawes et al., 2000; Bencze et al., 2018; Potter and Burgess, 2022). cAMP-PKA is a major cellular signaling pathway that is involved in the regulation of depression (Wang et al., 2018; Gao et al., 2022). Several studies have reported that alterations of the cAMP-PKA signaling pathway affect both TH and DBH expression. The direct activation of cAMP induces the upregulation of neuronal TH expression (Sklair-Tavron et al., 1994; Chen et al., 2008). The phosphorylation of PKA positively regulates the catalytic activity of TH (Lehmann et al., 2006). cAMP-dependent protein kinase regulates the transcription of DBH (Chen et al., 2008). Interference with cAMP-mediated DBH regulation may be involved in the etiology of anxiety and depression symptoms (Kim et al., 2014). Overall, inhibition of the cAMP-PKA pathway may cause the downregulation of TH and DBH, thereby impairing NE synthesis. This may explain our experimental findings that the acute microinjection of MCH in the LC significantly downregulated cAMP and p-PKA levels and the expression of both TH and DBH. Pretreatment with an MCHR1 antagonist significantly restored cAMP-PKA signaling and upregulated the expression of TH and DBH. These results are mostly consistent with the noradrenergic alterations that were observed in the CUS suffered rats. MCH may act on MCHR1 on noradrenergic neurons to inhibit downstream cAMP-PKA signaling and subsequently reduce TH and DBH expression, ultimately reducing NE synthesis. This may be an underlying mechanism by which abnormal activity of the MCH system affects NE synthesis. However, the exact mechanism by which decreased synthesis and transmission of NE is involved in regulating depression-like behaviors remains unclear. MCH has a stimulatory effect on the HPA axis (Bluet-Pajot et al., 1995; Kennedy et al., 2003), and our previous study confirmed that antagonizing MCHR1 in the LC reversed depression-like behaviors induced by hyperactivation of HPA axis (Ye et al., 2018). It has been reported that stress-induced hyperactivity of the LC leads to activation of the HPA axis and further stimulates α_2_ auto receptors in LC-NE neurons (Kobilka 2011). Therefore, we speculate cautiously that in the chronic stress condition, MCH activate HPA axis, which in turn activates inhibitory a_2_ receptors in the LC, thereby decreasing the synthesis and release of NE in downstream brain regions, mPFC in this case, and participating in the regulation of depressive-like behaviors.

Our present study is still inadequate, there are limitations associated with LC tissue collection, microinjection and other approaches needs further improvement. Besides, future studies need to directly activate the LC-MCH system to observe its effect on NE level and depression-like behavior would be more convincing.

In conclusion, we found that CUS-induced changes in MCH/MCHR1 levels in the LH and LC varied with different stress durations, and MCHR1 antagonism improved depression-like behaviors, suggesting that the MCH system in the LC is indeed closely related to the formation of depression-like behaviors. These CUS duration-relating changes occurred in MCH system and its association with depression-like behaviors were first observed in the present study. Our results also suggest that MCH and MCHR1-mediated cAMP-PKA signaling may regulate noradrenaline producing enzymes in the LC and noradrenergic dysregulation may underlie the occurrence of depression-like behaviors. These findings have contributed to the understanding of how MCH participates in noradrenergic regulatory networks in the brain and provided a new basis for the correlational pathogenesis of depression.

## Supplementary Material

pyad069_suppl_Supplementary_Tables_S1_Figures_S1-S3

## Data Availability

The data underlying this article will be shared on reasonable request to the corresponding author.
