# Supplementary material for: Chronic Stress-Induced Elevation of Melanin-Concentrating Hormone in the Locus Coeruleus Inhibits Norepinephrine Production and Associated With Depression-Like Behaviors in Rats
Source: Int J Neuropsychopharmacol. 2023 Dec 22;27(1):pyad069. doi: 10.1093/ijnp/pyad069 (PMC10799331; doi:10.1093/ijnp/pyad069)
Supplement: pyad069_suppl_Supplementary_Tables_S1_Figures_S1-S3 [file pyad069_suppl_supplementary_tables_s1_figures_s1-s3.docx]

**Chronic stress-induced elevation of melanin-concentrating hormone in the locus coeruleus inhibits norepinephrine production and associated with depression-like behaviors in rats**

Nurhumar Kurban, M.M ^1^; Yu Qin, B.S ^1^; Hui-Ling Zhao, B.S ^1^; Xiao Hu, PhD ^1^; Xi Chen, B.S ^1^; Yi-Yi Zhao, B.S ^1^; Yu-Shuo Peng, B.M ^1^; Hong-Bo Wang, PhD ^2^; Su-Ying Cui, PhD ^1, 2,^ *; Yong-He Zhang, PhD^1,^ *

^1^ Department of Pharmacology, Peking University, School of Basic Medical Science, Beijing 100191, China.

^2^ Key Laboratory of Molecular Pharmacology and Drug Evaluation, Ministry of Education, Yantai University, Yantai 264005, China

***Correspondence**:

Su-Ying Cui, PhD, [csy@bjmu.edu.cn](mailto:csy@bjmu.edu.cn);

Yong-He Zhang PhD, [zhyh@hsc.pku.edu.cn](mailto:zhyh@hsc.pku.edu.cn);

Department of Pharmacology, Peking University, School of Basic Medical Science, Beijing 100191, China.

**Supplementary Table 1. CUS Procedure.**

The rats were exposed to 12 different stressors (two stressors per day), including four types of strong stressors (footshock, tail pinch, body restriction, and ice-water bath) and eight types of mild stressors (bright light stimulation, water or food deprivation, white noise, cage shaking, wet cage, crowded housing, tilted cage, and inverse light/dark cycle). The specific CUS process is provided in Table 1.

Table 1. CUS Procedure.

|  | CUS 7 | CUS 14 | CUS 21 | CUS 28 |
| --- | --- | --- | --- | --- |
| Day 1 |  |  |  | White noise  Tilted cage |
| Day 2 |  |  |  | Crowded housing  Ice-water bath |
| Day 3 |  |  |  | Tail pinch  Water deprivation |
| Day 4 |  |  |  | Wet cage  Food deprivation |
| Day 5 |  |  |  | Inversed day cycle  Footshock |
| Day 6 |  |  |  | Body restriction  Light stimulation |
| Day 7 |  |  |  | Dark illumination Cage shaking |
| Day 8 |  |  | Crowded housing  Body restriction | Crowded housing  Body restriction |
| Day 9 |  |  | White noise  Tail pinch | White noise  Tail pinch |
| Day 10 |  |  | Food deprivation  Night illumination | Food deprivation  Night illumination |
| Day 11 |  |  | Light stimulation  Footshock | Light stimulation, Footshock |
| Day 12 |  |  | Tilted cage  Reversed day cycle | Tilted cage  Reversed day cycle |
| Day 13 |  |  | Wet cage  Water deprivation | Wet cage  Water deprivation |
| Day 14 |  |  | Cage shaking  Ice-water bath | Cage shaking  Ice-water bath |
| Day 15 |  | White noise  Light stimulation | White noise  Light stimulation | White noise  Light stimulation |
| Day 16 |  | Food deprivation  Ice-water bath | Food deprivation  Ice-water bath | Food deprivation  Ice-water bath |
| Day 17 |  | Wet cage  Crowded housing | Wet cage  Crowded housing | Wet cage  Crowded housing |
| Day 18 |  | Cage shaking  Tail pinch | Cage shaking  Tail pinch | Cage shaking  Tail pinch |
| Day 19 |  | Footshock  Night illumination | Footshock  Night illuminatio | Footshock  Night illuminatio |
| Day 20 |  | Tilted cage  Water deprivation | Tilted cage  Water deprivation | Tilted cage  Water deprivation |
| Day 21 |  | Inversed day cycle  Body restriction | Inversed day cycle  Body restriction | Inversed day cycle  Body restriction |
| Day 22 | Night illumination  Wet cage | Night illumination  Wet cage | Night illumination  Wet cage | Night illumination  Wet cage |
| Day 23 | Tail pinch  Crowded housing | Tail pinch  Crowded housing | Tail pinch  Crowded housing | Tail pinch  Crowded housing |
| Day 24 | Body restriction  White noise | Body restriction  White noise | Body restriction  White noise | Body restriction  White noise |
| Day 25 | Food deprivation  Inversed day cycle | Food deprivation  Inversed day cycle | Food deprivation  Inversed day cycle | Food deprivation  Inversed day cycle |
| Day 26 | Footshock  Light stimulation | Footshock  Light stimulation | Footshock  Light stimulation | Footshock  Light stimulation |
| Day 27 | Ice-water bath  Tilted cage | Ice-water bath  Tilted cage | Ice-water bath  Tilted cage | Ice-water bath  Tilted cage |
| Day 28 | Pre-swim (15min)  Water deprivation | Pre-swim (15min)  Water deprivation | Pre-swim (15min)  Water deprivation | Pre-swim (15min)  Water deprivation |

**Supplementary Fig. 1. β-actin expression**

In the western-blot test, the target protein expression was normalized to β-actin expression and the data were expressed as “Fold change over control or vehicle group %”. According to our results, stress or treatments alone did not impact β-actin levels. In detail, 7, 14, 21 or 28 days of CUS duration did not affect β-actin level in the first experiment (F _(4, 35)_ = 0.1919, p > 0.05, Supplementary Fig. 1A). In the second experiment, 28 days of CUS (F _(1, 20)_ = 1.991, p > 0.05) or 7 days of SNAP treatment (F _(1, 20)_ = 0.3514, p > 0.05) did not affect β-actin level (Supplementary Fig. 1B). In the third experiment, MCH microinjection (F _(1, 20)_ = 0.1630, p > 0.05, Supplementary Fig. C; F _(1, 20)_ = 0.6084, p > 0.05, Supplementary Fig. 1D) or SNAP pretreatment (F _(1, 20)_ = 0.41544, p > 0.05, Supplementary Fig. 1C; F _(1, 20)_ = 0.0448, p > 0.05, Supplementary Fig. 1D) did not impact β-actin level.


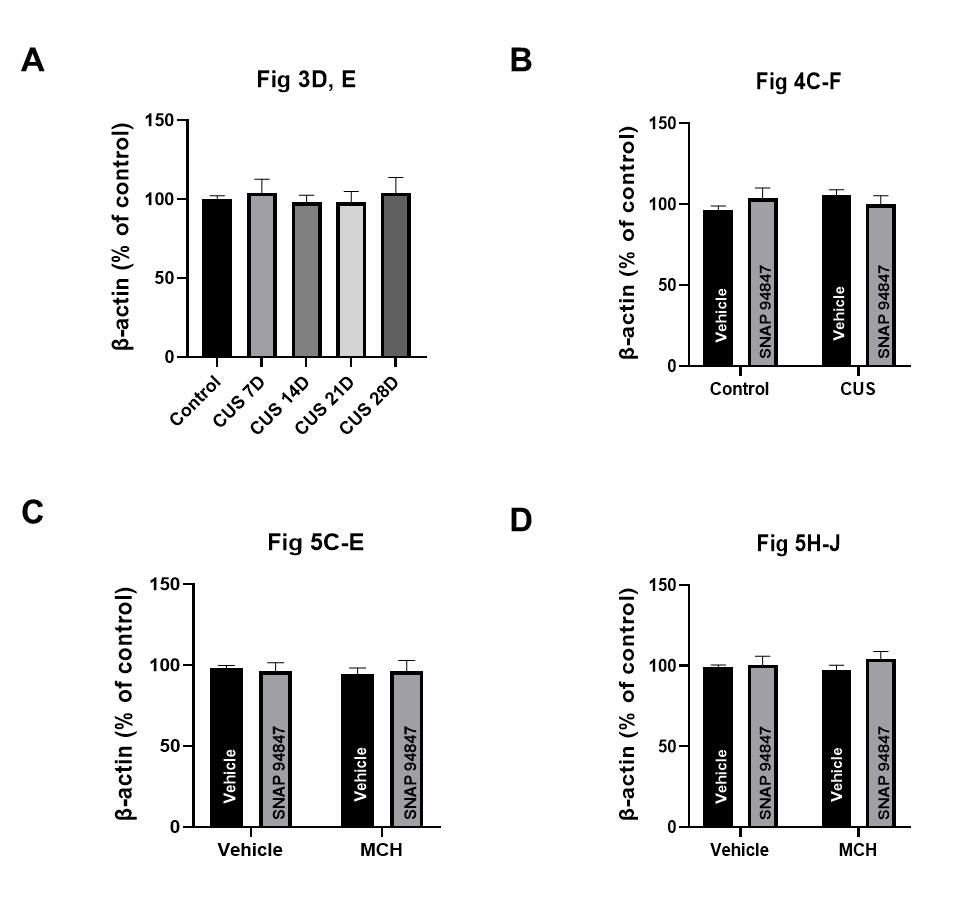


**Supplementary Fig1. Stress or drug treatments alone did not impact β-actin levels.** (A) Western blot analysis of β- actin expression during different CUS duration. (B) Western blot analysis of β- actin expression under CUS or SNAP-94847 microinjection. (C, D) Western blot analysis of β- actin expression under MCH or SNAP-94847 microinjection. The data are expressed as the mean ± SEM (one-way or two-way ANOVA followed by Newman-Keuls *post hoc* test).

**Supplementary Fig. 2. Cannula misplacement data of Experiment 2.**

In the CUS experiment (experiment 2), as we described in the *Result*, CUS significantly induced depression-like behavior, and the microinjection of SNAP-94847 in the LC significantly reversed CUS-induced depression-like behavior without significant alteration of total fluid intake in sucrose preference test and total traveled distance in locomotor test (*Fig 4, B1-B4 in the manuscript,* Supplementary Fig. 2A-D). However, SNAP-94847 (or Vehicle) microinjection outside the LC in the CUS-suffered rats, immobility time (F _(1, 20)_ = 1.040; p > 0.05, Supplementary Fig. 2A), sucrose preference (F _(1, 20)_ = 0.0010; p > 0.05, Supplementary Fig. 2B) total fluid intake (F _(1, 20)_ = 0.0020; p > 0.05, Supplementary Fig. 2C) and total traveled distance (F _(1, 20)_ = 0.1060; p > 0.05, Supplementary Fig. 2D) did not alter significantly, indicating SNAP-94847 microinjection outside the LC did not reversed CUS-caused depression-like behavior (Supplementary Fig. 2A-D).

.
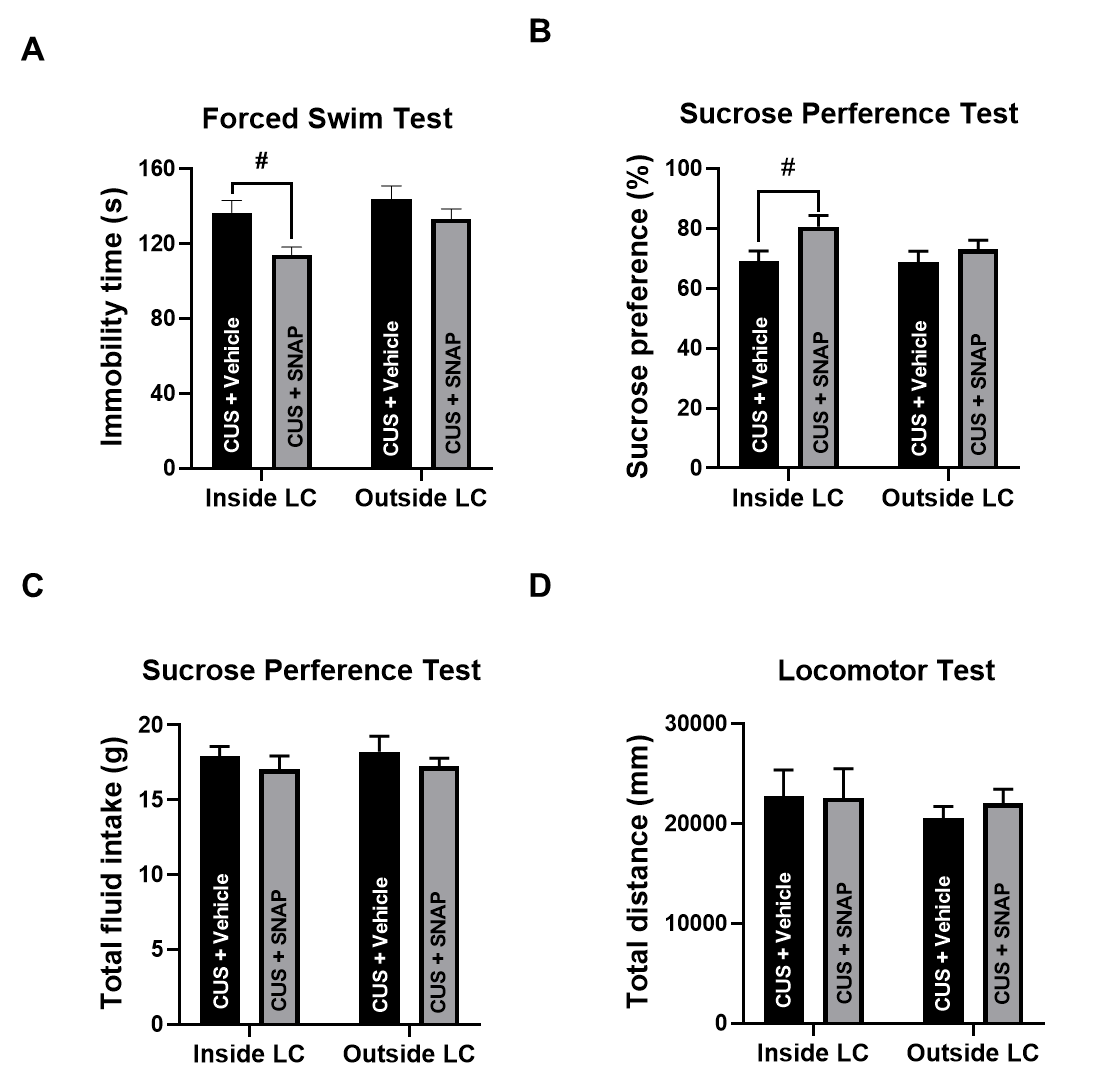


**Supplementary Fig 2. (A-D) SNAP-94847 microinjection outside the LC did not reverse CUS-caused depression-like behavior.** Immobility time in the FST (A), sucrose preference (B) and total fluid intake (C) in the SPT, and total distance traveled in the locomotor test (D) were evaluated (n1, n2, n3 n4= 7, 7, 5, 5). The data are expressed as the mean ± SEM. ^#^p < 0.05 and ^##^p < 0.01 *vs.* CUS+Vehicle-Inside LC group. (two-way ANOVA followed by Newman-Keuls *post hoc* test).

**Supplementary Fig. 3. Cannula misplacement data of Experiment 3.**

In MCH experiment (experiment 3), as we described in the *Result*, the intra-LC microinjection of MCH induced depression-like behavior. Pretreatment with SNAP-94847 in the LC blocked MCH-induced depression-like behaviors, without significant alterations of the total traveled distance in the locomotor test and total fluid intake in the SPT (Fig 5, B1-B4 in the manuscript, Supplementary Fig. 3A-D).

However, we found that, there were significantly differences in these behavior data between Vehicle+MCH-Inside LC group and Vehicle+MCH-Outside LC group. Compared to Vehicle+MCH-Inside LC group, MCH treated outside the LC did not exhibit depression-like behavior in forced swim (F _(1, 25)_ = 3.809; p < 0.05, Supplementary Fig. 3A) and sucrose preference tests (F _(1, 25)_ = 9.842; p < 0.05, Supplementary Fig. 3B). Meanwhile, no significant behavioral changes observed in immobility time (F _(1, 25)_ = 1.660; p > 0.05, Supplementary Fig. 3A), sucrose preference (F _(1, 25)_ = 2.736; p > 0.05, Supplementary Fig. 3B) total fluid intake (F _(1, 25)_ = 0.1546; p > 0.05, Supplementary Fig. 3C) and total traveled distance (F _(1, 25)_ = 0.03457; p > 0.05, Supplementary Fig. 3D) with SNAP-94847 pretreatment rats with injection site outside the LC (Supplementary Fig3. A-D).


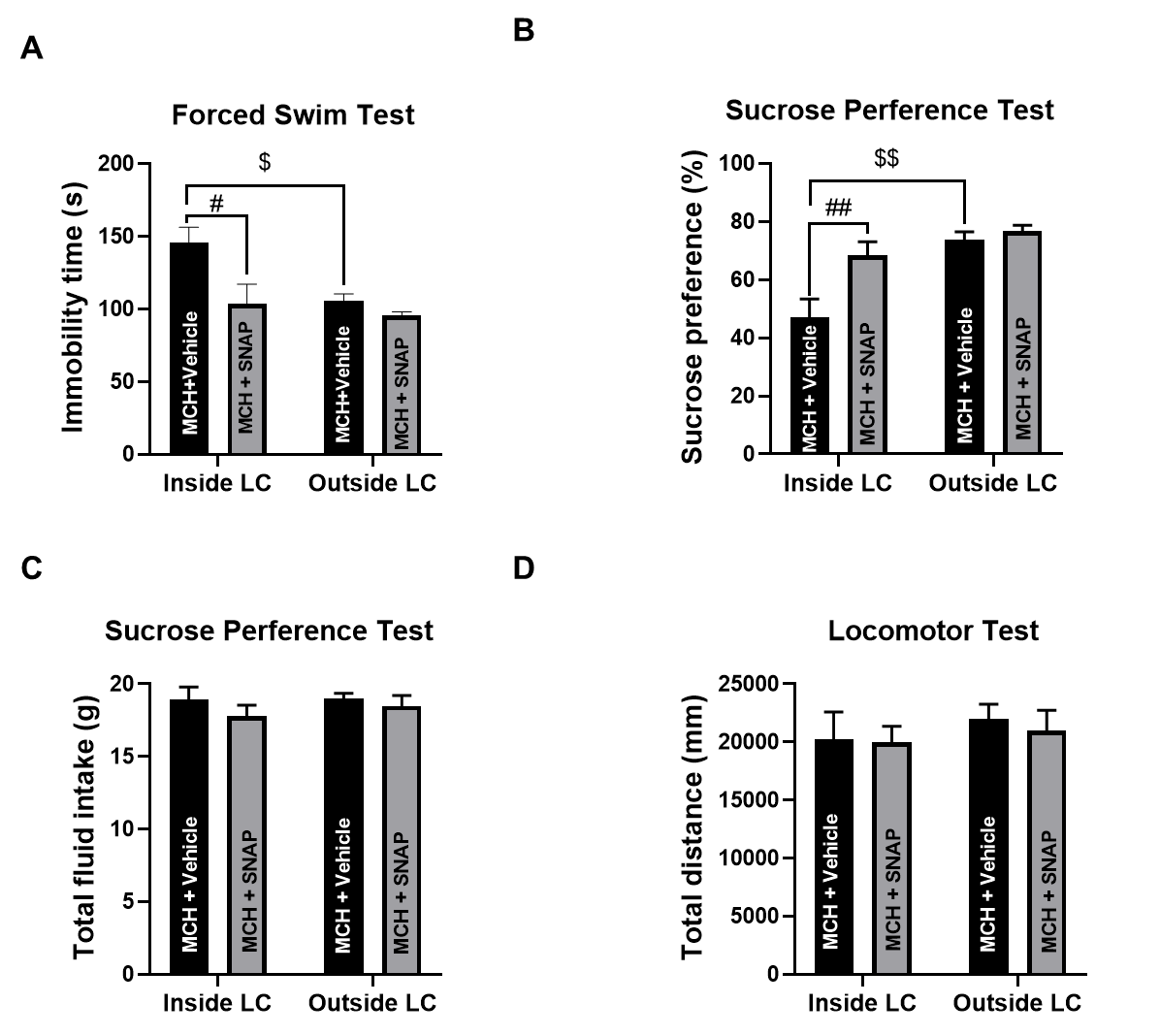


**Supplementary Fig. 3. (A-D)** **Whether SNAP-94847 or MCH microinjection did not alter the behavioral changes in rats with injection site outside the LC.** Immobility time in the FST (A), sucrose preference (B) and total fluid intake (C) in the SPT, and total distance traveled in the locomotor test (D) were evaluated (n1, n2, n3, n4 = 9, 10, 5, 5).The data are expressed as the mean ± SEM. ^#^p < 0.05 and ^##^p < 0.01 *vs.* Vehicle+MCH-Inside LC group; ^$^p < 0.05 and ^$$^p < 0.01*vs.* Vehicle+MCH-Outside LC group (two-way ANOVA followed by Newman-Keuls *post hoc* test).
